# Development of an IoT Electrostimulator with Closed-Loop Control

**DOI:** 10.3390/s22093551

**Published:** 2022-05-07

**Authors:** Túlio Fernandes De Almeida, Luiz Henrique Bertucci Borges, André Felipe Oliveira de Azevedo Dantas

**Affiliations:** Graduate Program in Neuroengineering, Edmond and Lily Safra International Institute of Neuroscience, Santos Dumont Institute, Av. Alberto Santos Dumont, 1560, Zona Rural, Macaiba 59280-000, RN, Brazil; tuliofalmeida@hotmail.com (T.F.D.A.); luiz.borges@edu.isd.org.br (L.H.B.B.)

**Keywords:** rehabilitation, closed-loop control, electrostimulation, sensors in healthcare, medical device

## Abstract

The most used approach in the motor rehabilitation of spinal cord injury is functional electrical stimulation. However, current devices do not provide real-time feedback, work in the closed-loop, and became remotely operable. In this scenario, this paper presents the development of an open access 4-channel IoT electrostimulator device with an inertial sensor. The electrostimulator circuit was designed with four modules: Boost Converter, H-bridge, Inertial Measurement Unit, and Processing Module. The firmware was implemented in the processing module to manage the modules to perform closed-loop stimulation (using PID controller). To perform the proof of concept of the device, a closed loop test was performed to control the ankle joint, performing the movements of dorsiflexion, plantar flexion, inversion, and eversion. The designed hardware allowed one to freely change the boost converter voltage and modulate the signal with 200 μs of pulse duration and 50 Hz of period in a safe and stable way. Furthermore, the controller was able to move the ankle joint in all desired directions following the reference values and respecting the imposed constraints. In general, the developed hardware was able to safely control a closed-loop joint.

## 1. Introduction

Spinal cord injury (SCI) is the most disabling condition for humans, usually caused by traumatic accidents (e.g., traffic accidents, falls) or violence [1,2]. SCI led to severe impairment in the sensory, motor, and autonomous systems below the injury level, and secondary complications such as chronic pain and bladder and bowel dysfunction, along with increased susceptibility to respiratory and heart problems [1,3]. In this sense, the financial burden for the health system is immense, affecting the patient, insure companies, hospitals, and government [3,4].

To reduce the financial impact and improve the quality of life of people with SCI, the development of new rehabilitation techniques and medical devices is essential. The most used approach in the motor rehabilitation of SCI is functional electrical stimulation (FES) [5,6,7], which uses electrical current to cause a muscle to contract and promote functional improvement by increasing muscle strength and range of movement [8]. However, commercial FES equipment has not been adapted to the current Internet of Things (IoT) scenario [5,7]. The currently available devices do not have access to databases, integrated control systems, and real-time feedback [9], there are only a few devices developed or adapted in laboratories that are not open access [10].

The most complex FES applications in rehabilitation are assisted cycling and walking. Based on the actual devices, the execution of these cyclic movements is performed based on time or by devices attached to FES equipment [6,11,12]. To increase the effectiveness of the therapy with FES, it should provide real-time feedback and work in the closed-loop. In addition, it is necessary to democratize access to this type of equipment, to promote the emergence of new techniques and rehabilitation protocols [9]. The system presented here aims to meet these conditions, providing an open IoT electrostimulator device.

This study aimed to (1) develop a low-cost IoT electrostimulator, (2) present a closed-loop functionality using inertial sensors, and (3) perform a proof-of-concept of the proposed electrostimulator by a closed-loop experiment to control the ankle joint.

## 2. Materials and Methods

**Figure 1:**
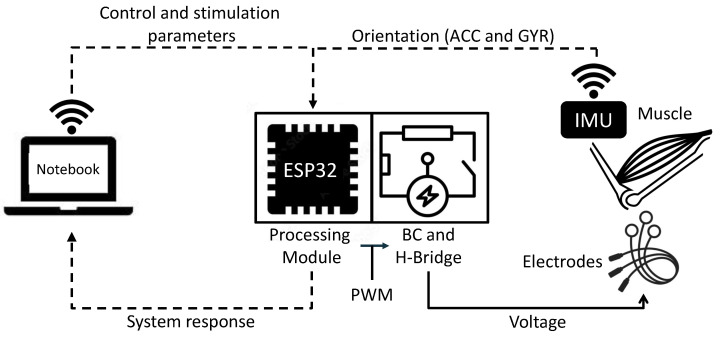
Functional diagram of the proposed system.

In this section, there are material description, components, concepts, and techniques to develop a 4-channel IoT electrostimulator device. This project was approved by the ethics committee of Santos Dumont Institute under the protocol 53127921.2.0000.0129 approved in 23 December 2021. The functional diagram of the system is depicted in Figure 1.

As shown in Figure 1, the setup is composed of a 4-channel Fes (processing module, BC, H-bridge, and IMU), a notebook to configure the FES and send the setpoint values and the controller, and the controlled system (muscle and joint angle). In this scheme, the notebook is responsible to start via Wi-Fi the FES and receive all generated data. After the stimulation module is started, it cyclically reads the sensors data, calculates the joint angles, compares them with the reference (error) sent from notebook, calculates the control actions and uses them to contract the muscles in the direction the error is minimized. In the following subsections, the building information of the hardware and firmware are detailed as well as the tests setup.

### 2.1. Hardware Design

**Figure 2:**
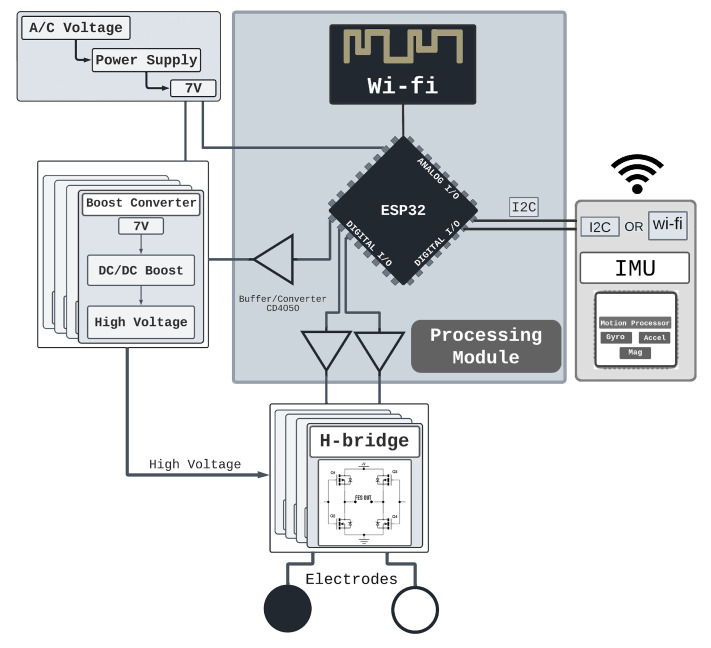
Hardware Architecture - The system was designed with a 7V power supply, 4 boost modules ad H-Bridges and an IMU sensor that gives feedback via I2C or Wi-Fi.

The electrostimulator circuit was designed with four modules: boost converter (BC), H-bridge, inertial measurement unit (IMU), and processing module, as shown in Figure 2.

**Figure 3:**
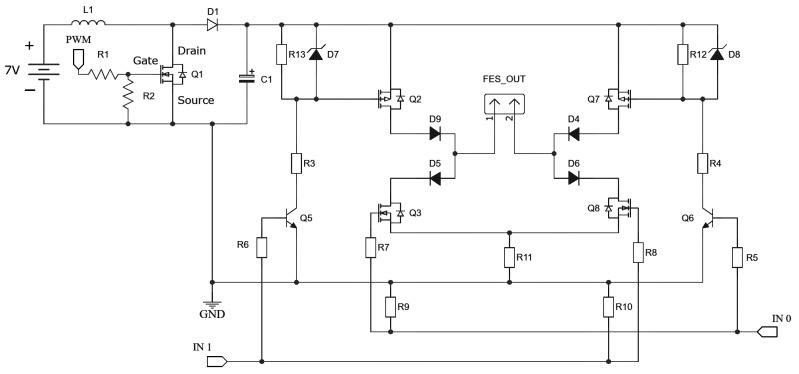
Electrostimulator circuit. The designed circuit has two parts. The first one is the boost that amplifies the 7 V input voltage accordingly to the PWM input in Q1. After the D1-C1 node, there is a connection from the boost to the H-Bridge.

The processing module (Figure 2), uses a microcontroller (ESP32-DevKitC [13]) to (1) communicate based on the Message Queuing Telemetry Transport (MQTT) protocol; (2) manage the stimulation signals using pulse width modulation (PWM) signals (used to amplify boost voltage and modulate the signal controlling the H-bridge); and (3) extract data from the IMU. The communication between the processing module and the IMU is based on the Inter-Integrated Circuit ($I^{2}C$) protocol [14] with an acquisition frequency of 100 KHz, allowing the use of any IMU. Here, the GY-80 (10 degrees of freedom—accelerometer, gyroscope, magnetometer and barometer) sensor was used due to previous experience in other projects. The H-bridge [15,16] and BC [15] are essential part of the device, and the circuits are presented in Figure 3.

Due to the need to consider multiple stimulation channels, user safety and generation of stimulation signals, the Processing Module have to manage the amplification and modulation circuits to operate up to 4 stimulation channels. These amount of channel can stimulate 4 muscles and perform functional movements using the IMU as reference.

IMU module was developed based on the work of [17], they developed the Joint Angle Measurement Acquisition (JAMA) device. JAMA is an open-source hardware capable of extracting and sending IMU data via Wi-Fi, and it was applied in the assessment of human movement. In this work, the objective was to use the human movement to create an closed-loop electrostimulator, using the IMU data to calculate control actions and produce functional movements (e.g., gait, pedaling). For this, it was performed some adaptations to integrate JAMA firmware with the electrostimulator routines.

### 2.2. Structure of the Control System

The ankle was the chosen joint since it has a fundamental role in maintaining stability during posture (regulatory) and helps the gait movement (setpoint tracking) [18]. To generate movement for a joint in each direction, two muscles (an agonist and antagonist pair) must be stimulated [19].

**Figure 4:**
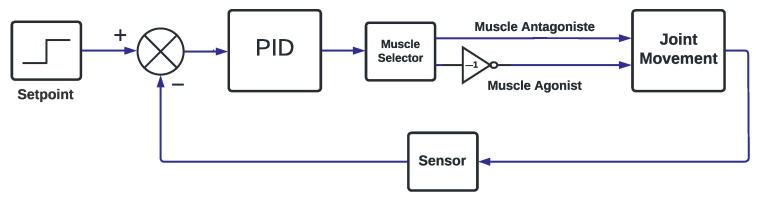
PID Block Diagram.

Control mechanisms that help maintain the body’s balance have been investigated. In a first simple control approach, two muscles shall be actuated as shown in Figure 4.

Figure 4 shows the scheme for controlling one angle in joint movement. As seen, to control the desired angle, an error signal must be generated from set point and output angle (calculated using accelerometer and gyroscope data fused with complementary filter [17]). After that, the controller calculates the control action (PID with Conditional Integration [20]). In sequence, the control action is directed to the right muscle (Muscle Selector) as a duty cycle for the boost, causing the stimulation amplitude necessary to correct the error. In the case when more than one angle have to be controlled, the angles in joint movement can be considered not coupled. In other words, the control design can be performed independently for each joint angle.

For its simple modeling and tuning effort [19], the chosen controller is the PID. According to [21] there are three reasons that make the PID so important: (a) track record of success; (b) wide availability; (c) simplicity of application. PID is based on three control actions (Proportional, Integral and Derived). By combining proportional, integral and derivative actions in a single controller, the PID formulation can be schematized as shown in the Equation (1) [22,23]:

(1) $$\begin{array}{c} u\left(t\right)=K_{p}e\left(t\right)+K_{i}\int_{0}^{t}e\left(t\right)dt+K_{d}\frac{de(t)}{dt} \end{array}$$

being that:

- u(t) corresponds to the control action (duty cycle) calculated on time *t*;
- $K_{p}$ is the proportional gain;
- $K_{i}$ is the integral gain;
- $K_{d}$ is the derivative time;
- e(t) is the difference between the desired angle and the joint angle;
- $\int_{0}^{t}e\left(t\right)dt$ is the integral of the error;
- $\frac{de(t)}{dt}$ is the derivative of the error.

Tuning is required for PID to be used, for ankle joint control, this tune needs to consider maintaining stability in regulatory and track gait movement. Additionally, the PID needs to ensure both stability and avoid pain for the experiment participant. This is an issue because, the more aggressive the controller is, the greater the discomfort for the subject.

### 2.3. Firmware Design

After the hardware and controller definition, its necessary to develop a firmware to integrate the device modules and implement the PID controller. There were developed two routines in C++ to control the device in Open-Loop and Closed-loop Stimulation modes and are described below:

1. Open-Loop Stimulation: it is a routine developed to activate electrostimulator channels (individually or together) to perform stimulation similar to conventional devices. In this routine, the user can determine the stimulation parameters: boost duty cycle (stimulus amplitude), pulse duration, and frequency.
2. Closed-Loop Stimulation: this routine will use an IMU as sensor to control the joint angle using the electrical stimulation. For this, the user must determine the IMU parameters: acquisition frequency (Hz) and data acquisition time (seconds). In addition to determining the sensor configurations, it is necessary to define the parameters ($K_{p}$, $K_{i}$, $K_{d}$ and output limits) of the 2 implemented PID (proportional-integral-derivative) controllers and the stimulation parameters (the same presented in Open-Loop Stimulation).

One of the most used closed-loop controllers in healthy applications is the Proportional-Integral-Derivative (PID), due to the simplicity and efficiency [24,25,26]. Here, the PID controllers were implemented in the Processing Module using C++. They use the following parameters: error ($K_{p}$), the integral of the error ($K_{i}$), and the derivative of the error ($K_{d}$) to calculate the stimulus voltage of 2 channels. Each channel is responsible for producing movement in one direction by stimulating muscle responsible for the desired movement (e.g., tibialis anterior for dorsiflexion). Furthermore, each PID is responsible for one degree of freedom, controlling the agonist and antagonist of the movement (e.g, on channel on the tibialis anterior for dorsiflexion, and the other channel on the gastrocnemius for the plantar flexion). Moreover, the C++ implemented PID saturate the control action. When this happens, the routine also stops integrating to avoid the windup phenomenon.

The implemented PID are configured so that the positive control signal stimulates the agonist muscle to increase the angle value and the negative control signal the antagonist muscle to decrease the joint angle. In this sense, the user must determine the PID parameters according to the placement of the electrodes. This was performed using an interactive Python script developed to control the ankle joint.

In addition to the firmware, it is necessary to develop a script to choose, configure and run the routines. For this, it were developed a script in Python using MQTT protocol to find the device with the ID of each channel/IMU and establish the Wi-Fi communication. Stimulation routines and the application script are available on GitHub (https://github.com/luizbertucciborges/fes4channels, accessed on 25 March 2022).

### 2.4. Structural Hardware Test

This section presents the design of tests to evaluate the performance and applicability of the electrostimulator. To evaluate the functioning of the BC and H-bridge modules, an oscilloscope was used to measure the PWM signals at the input of the boost circuit and the signal sent to the H-Bridge. Also, the ability of the BC to amplify the stimulation signal, and the H-Bridge to modulate it was also evaluated.

The test consisted of simulating a resistive load, changing the duty cycle from 0 to 5 and simultaneously sending a 50 Hz Hz modulation signal to the H-Bridge. Periodic and alternating stimulation was performed on the H-Bridge, alternating the stimulation every 200 μs with a 19.6 ms pause at each cycle. The logic of the test was to evaluate if the electrostimulator was able to maintain the frequency of the modulated signal of 50 Hz while increasing and decreasing the voltage in the BC module.

### 2.5. Closed-Loop Test

Closed-loop routine test was performed using the four stimulation channels to control the ankle joint as a proof of concept. Ankle joint was chosen because it is able to move in 3 directions (plantar flexion/dorsiflexion, eversion/inversion and adduction/abduction) and it is possible to passively replicate these movements using electrical stimulation. The electrodes were placed over the muscles: tibialis anterior (dorsiflexion), gastrocnemius (plantar flexion), peroneus longus and brevis (eversion), and flexor hallucis longus (inversion).

The test was performed on a healthy subject (woman, 1.55 m, 55 kg, Body Mass Index 22.9), and using the following stimulation parameters: frequency of 50 Hz, pulse duration of 200 μs, and amplitude (duty cycle) of 5% (0–100%). The stimulation parameters used and the location of the motor points were determined based on standard protocols [12], aiming at safety, decreasing the participant’s discomfort and stimulation efficiency. Discomfort/pain was measured using the Numeric Pain Rating Scale throughout the experiment, the participant was instructed on the functioning of the scale and whenever she feel it changed she should report [27]. The scale ranges from 0 to 10, with 0 being no pain/discomfort and 10 being the worst possible pain experience. It was previously established that pain/discomfort greater than or equal to 6 would stop the experiment, or at any time at the request of the participant.

**Figure 5:**
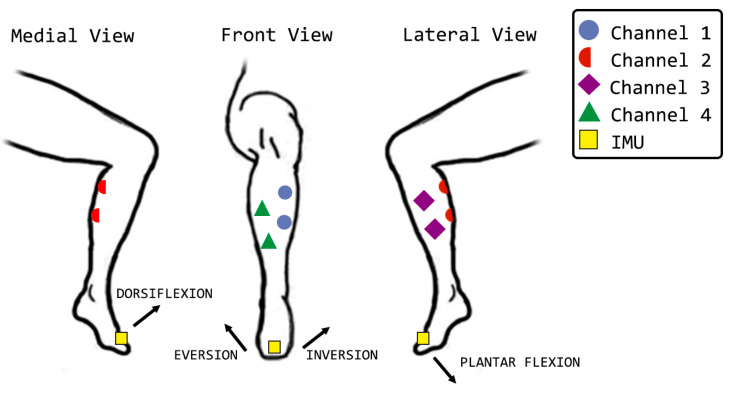
Setup test. Electrodes and IMU positing in medial, frontal, and lateral views. The figure shows electrodes of each channel positioning and indicates the direction of movements.

Before performing the data acquisition, the electrodes were positioned and removed to ensure their correct fit. In addition, other simpler tests were performed at earlier times (e.g., knee flexion and extension) to assess the system. Thus, the electrodes and the JAMA were positioned according with Figure 5.

PID tuning were performed using the step response [28], because the system (human body) is non-linear and the step response is bounded (which means we can use magnitudes that imply neither discomfort nor pain for the subject). The step response occurs with the tuning of the parameters ($K_{p}$, $K_{i}$, $K_{d}$) in order to start with less aggressive and end with more aggressive parameters, until a functional tuning was found [29,30]. This means that the PID controllers work “independently”, when a change in the setpoint of one PID does not influence the regulatory of the other. Therefore, the variation of the setpoint of one controller (responsible for eversion and inversion) does not change the orientation of the other (responsible for dorsiflexion and plantar flexion). Moreover, the electrostimulator parameters used were frequency of 50 Hz, pulse duration of 200 μs, and amplitude of 5% (0–100%). The control and stimulation parameters have been adjusted remotely and wireless.

This experiment was performed with the individual sitting with the lower limbs hanging. After tuning the controllers, the right foot was passively positioned in a neutral position using the electrostimulator, and then the movements of inversion, eversion, dorsiflexion, and plantar flexion of the ankle joint were performed sequentially.

## 3. Results

This section is intended to present the hardware, structural hardware test, and the closed-loop test.

### 3.1. Hardware

**Figure 6:**
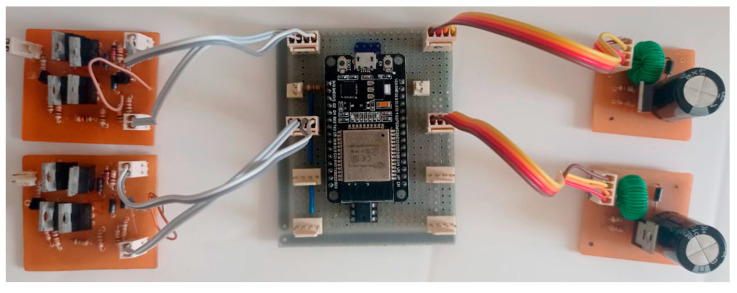
Electrostimulation built circuit. The figure shows the 2-channel circuit of the developed electrostimulator. On the left are two H-Bridge modules, in the center the processing module, and on the right, the boost converter.

The hardware was built as designed, with 4 channels, max voltage of $\pm 100\;V_{max}$ (9 W of power supply), inertial sensor feedback and 2.4 Ghz Wi-Fi communication. Moreover, the user can easily configure the channels of the application (add or remove) because the system is modular. The used power supply is a commercial one, so it matches the standardized recommendations. In addition, if the therapy requires more current, it is possible to change the power supply. Figure 6 shows the built circuit.

As seen in Figure 6, the processing module is the central part of the circuit. It allows the easy connection of 4 boosts and 4 H-Bridges using the properly connectors (in the figure, only 2 channels are shown due to the dimensions of the photo).

### 3.2. Structural Hardware Test

**Figure 7:**
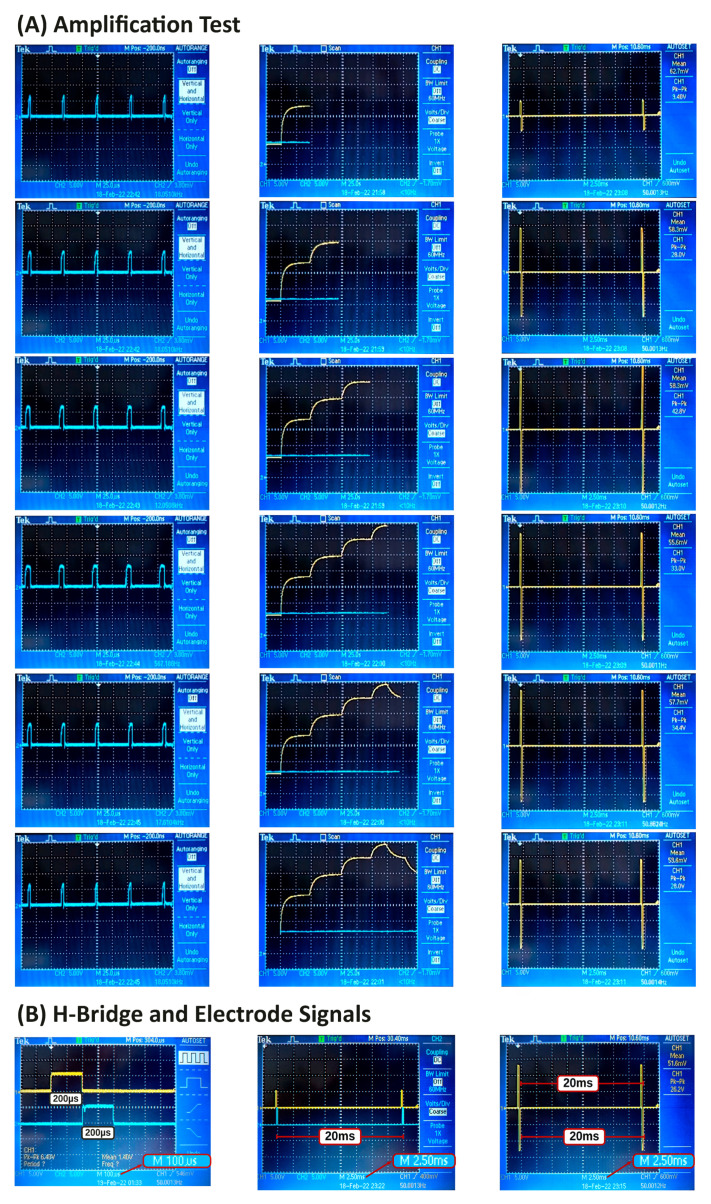
Structural Hardware Test. The figure shows the results of the structural test using an oscilloscope. (**A**) First column: PWM input to the Boost Control and H-Bridge. Second column: BC response to the PWM input signal. Third column: H-Brige response to the modulation signal. (**B**) Left: Two-channel BC response to PWM signal in 100 μs window. Center: The 2.5 ms reading window of the Two-channel BC. Right: The stimulation signal sent to the electrode.

Figure 7A present the results of the structural hardware test in columns. First column shows the PWM input to BC module; the second and third columns shows the response to the PWM signal by BC and H-Bridge respectively. According with the oscilloscope readings, the electrostimulator is stable to variations in the PWM signal, managing to maintain the frequency of 50 Hz throughout the test. To ensure this, the test was performed for five minutes three times without changing the results. Figure 7B shows the output of the H-Bridge, being able to maintain the 200 μs pulse duration and the 20 ms period. The figure shows some views of the signal using different time resolutions. The left image highlights the pulse duration in positive and negative phases, the center image shows the reading signal period with two oscilloscope channels and the right image shows the stimulation signal read at the electrode output.

### 3.3. Closed-Loop Stimulation

**Figure 8:**
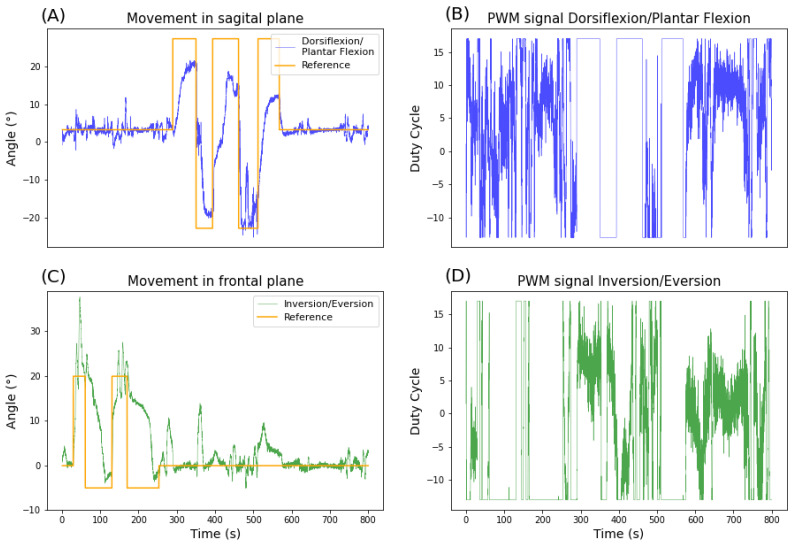
Closed-loop ankle joint control. (**A**) The position of the ankle joint (in degrees) in relation to the reference in sagital plane. (**B**) PWM signal for stimulation of stimulation channels to produce dorsiflexion and plantar flexion movements. (**C**) The position of the ankle joint (in degrees) in relation to the reference in frontal plane. (**D**) PWM signal for stimulation of stimulation channels to produce inversion and eversion movements.

The experiment described above worked successfully, using that electrode configuration and the device it was possible to control the ankle joint performing the movements of dorsiflexion, plantar flexion, inversion and eversion. The results of the experiment are shown in Figure 8.

As described in Section 2, before tuning the controller it is necessary to place the electrodes and verify the acceptable PWM/Voltage level. This procedure is specific for each application, as it is related to the individual’s characteristics (e.g., body mass and stimulus tolerance). After that, the controller must be tuned, using the step response with a gradual increase in aggressiveness, and the parameters obtained from step response are shown in the Table 1. Note that the objective of this experiment was the functional outcome, the possibility to control the ankle joint, not to perform the best tuning of the controller.

Figure 8A,C present the protocol described in the methodology, starting with the positioning of the foot in a neutral position (0 degrees in the first seconds), performing the inversion and eversion (Figure 8C), and ending with dorsiflexion and plantar flexion (Figure 8A). The JAMA signal is expected to track the reference signal (orange line) but not overlap at all times. Due to the type of tuning performed, the purpose of the test was to certify that the controller can produce the desired movements in a comfortable and safe way, so the tuning was done very slowly to avoid aggressive responses from the controller. In addition, the experiment was performed with a healthy individual, who even without moving, there are reflex responses and a certain level of basic muscle tone, and for the duration of the experiment may have tired and made minor adjustments. It is important to point out that throughout the experiment, the individual’s discomfort/pain remained between 0–2 on the Numeric Pain Rating Scale, which indicates a small discomfort that is expected.

The controller was able to execute the proposed movements, achieving the desired range of motion, 20° of dorsiflexion and plantar flexion, 20° of inversion and 5° of eversion (small negative deflections in Figure 8C). Since eversion was voluntarily difficult to perform in this condition, we opted for a smaller range of motion and performed for a shorter time to avoid producing pain in the participant. In addition, the positioning of the JAMA did not present good angular variation for eversion, which may have reduced the visualization of the angle obtained.

Figure 8B,D shows the PWM signals used to produce electrical stimulus. The PWM signals achieved the expected pattern. Because of the motor threshold (deadzone) and use of two controllers at the same time it was expected the regulatory control actions to have high variations, oscillating when the reference is 0. Additionally, when the ankle angles in both directions were forced to outside the physical range (determined beforehand), the controller saturated the control actions to the limits shown in Table 1. Because of that, sometimes, the controller do not reach the reference showing the controllers safety.

## 4. Discussion

### 4.1. Hardware

This paper presented a 4-channel open access IoT electrostimulator. Unlike other works [7,12,31,32] that introduce applications or some part of hardware [33,34], here the hardware of an IoT electrostimulator was presented and made available along with the firmware and how to build it.

The device presented here showed promising results as the controller performed well in a complex task respecting the imposed constraints. Usually the electrostimulator rely only on the H-bridge [33,34]. However, to increase the safety of the device (circuit and subject integrity), protection diodes capable of preventing reverse current or voltage were implemented. This is important because the electrostimulator has four channels and modulating signals simultaneously at different time intervals associated with different boost output voltage values, can cause negative voltages from one channel to another. In this sense, the diodes (D9, D4, D5 and D6) shown in Figure 3 are intended to prevent this situation, making the device safer.

A limitation of the electrostimulator in the actual version is the absence of an graphic interface that facilitates the manipulation of parameters and the creation of routines, which would expand the contexts of applicability (e.g., clinical research). Currently, the electrostimulator can be easily integrated into research laboratories. In addition, as the electrostimulator is open access, the community can develop a more robust system with more resources (modules, features, scripts and others).

An important factor to consider in open access devices is cost [17]. In this sense, the electrostimulator proposed in this work is low cost and its components are basic and accessible. The most expensive devices are the microcontroller (ESP32 costs 10 dollars) and the IMU (GY-80 costs 25 dollars, but another sensor can easily be adapted). The complete description of the components and how to build the circuit is available on GitHub (https://github.com/luizbertucciborges/fes4channels, accessed on 25 March 2022).

### 4.2. Proof of Concept

As seen in Table 1, the controller used is a PI, with the derivative term equal to zero. That happened because in the PID implementation there is no derivative filter and because the noise and some nonlinearities, the additions of this term increases the control signal variation which leads to participant discomfort and pain. On the other hand, the pure P controller was also tested, but because of the sensibility to participant’s positioning, the regulatory and tracking problem control loses performance. This loss of performance is directly associated with the disturbance rejection and the time constant of the systems [35].

The controllers with the sowed parameters presented good performance together with the electrostimulator, the controllers were designed in regulatory and tracking mode and were able to control the ankle joint (a multivariate system) respecting the output constraints. Constraints in this type of application are essential, as human joints have an articular system (bones, tendons, ligaments and capsule) to prevent injuries. In case of excessive movement in healthy individuals, the body’s response is pain and the withdrawal reflex [36]. In this sense, Figure 8 makes it possible to observe the good performance of the controllers.

Considering that PID controllers had to deal with saturation (limits of ankle joint angles), dead zone (muscle motor threshold) and hysteresis (in BC capacitor charges faster than discharges), non-linear muscle behavior, noise of JAMA measurement and perturbations (subject’s reactions to stimulation), they presented a good result, being able to maintain the setpoint and tracking the reference signal. In other words, the dead zone may lead to the chattering [37] seen in regulatory, some sensibility to subject position on the chair may cause some variation in mean magnitude of the control signal and the boost hysteresis may lead to a greater time constant when decreasing the voltage.

The non-linear behavior of the muscle [38] together with the characteristic of recruitment by electrical stimulation [30], are obstacles to the external control of the movement. Due to these factors and the simplicity of the PID, the result found is good and in case of a better tuning using auxiliary algorithms it can improve it [25].

In addition to simplicity, PID controllers are the most used [26]. More advanced control methods (e.g., model predictive control and deep learning based) tend to present better results, but they are more difficult to tune, computationally expensive and, in the case of human articulation, need a model otherwise they can neither predict the system behavior nor respect the constraints [7,23,39,40]. Thus, aiming to develop an open access electrostimulator system associated with closed-loop control, the PID controller was chosen. However, in the future, stimulation routines will be implemented for specific situations (e.g., cycling) with more advanced controllers.

### 4.3. Limitations and Perspectives

This study focused on the development of an IoT electrostimulator in its proof of concept of closed-loop operation in a healthy individual. For device validation, tests in other populations (e.g., people with spinal cord injury or stroke) must be performed, along with a functional use protocol (e.g., cycling and gait).

The test of the device made in closed-loop for ankle joint control, was carried out to proof the concept of the implemented control system. The experiment did not aim to achieve the best tuning of the controllers, but only the execution of the desired movements without producing pain or discomfort to the subject. In this way, other controller algorithms will be implemented in future to evaluate which one obtains the best performance with the electrostimulator.

Although the electrostimulator has worked stably and safely, and the controller has met expectations, it is possible to optimize the presented device (circuit and communication) to increase its robustness. Another important factor is the implementation of a GUI (graphical user interface), to facilitate its use in applications outside the laboratory and the development of more complex stimulation routines.

## 5. Conclusions

This article proposed an IoT electrostimulator together with a proof of concept of its closed-loop operation using PID controllers. The controllers were able to move the ankle joint in the proposed movements without generating pain or discomfort for the subject. In general, the developed hardware was able to safely control a closed-loop joint. In future work, we plan to implement other control algorithms and develop more tests in different situations.

## Figures and Tables

**Table 1:** PID parameters for ankle joint control.

Controller	Parameters	Values
PID 1	$K_{P}$	8.2
	$K_{I}$	3.8
Dorsiflexion/	$K_{D}$	0
Plantar Flexion	Min Limit	−14
	Max Limit	10
PID 2	$K_{P}$	8.1
	$K_{I}$	3.2
Inversion/	$K_{D}$	0
Eversion	Min Limit	−12
	Max Limit	14

## Data Availability

All codes and the tutorial on how to build the device are available on GitHub (https://github.com/luizbertucciborges/fes4channels, accessed on 25 March 2022). In addition, the device used to measure the joint angle (JAMA) is available on GitHub (https://github.com/tuliofalmeida/jama, accessed on 25 March 2022).
